# Transdisciplinarity of India’s master’s level public health programmes: evidence from admission criteria of the programmes offered since 1995

**DOI:** 10.1186/s12960-022-00713-4

**Published:** 2022-02-02

**Authors:** Kumaravel Ilangovan, Sendhilkumar Muthappan, Keerthiga Govindarajan, Vignesh Vairamani, Vettrichelvan Venkatasamy, Manickam Ponnaiah

**Affiliations:** ICMR-National Institute of Epidemiology, Chennai, Tamil Nadu 600077 India

**Keywords:** Public health, One health, Education, Public health practice, Interdisciplinary placement, Universities, Accreditation, Health systems, Health workforce

## Abstract

**Introduction:**

In the Indian subcontinent, Master’s-level Public Health (MlPH) programmes attract graduates of diverse academic disciplines from health and non-health sciences alike. Considering the current and futuristic importance of the public health cadre, we described them and reviewed their transdisciplinarity status based on MlPH admissibility criteria 1995 to 2021.

**Methods:**

Using a search strategy, we abstracted information available in the public domain on MlPH programmes and their admissibility criteria. We categorized the admission criteria based on specified disciplines into Health science, Non-health science and Non-health non-science categories. We described the MlPH programmes by location, type of institution, course duration, curriculum, pedagogical methods, specializations offered, and nature of admission criteria statements. We calculated descriptive statistics for eligible educational qualifications for MlPH admission.

**Results:**

Overall, 76 Indian institutions (Medical colleges—21 and Non-medical coleges—55) offered 92 MlPH programmes (Private—58 and Public—34). We included 89 for review. These programmes represent a 51% increase (*n* = 47) from 2016 to 2021. They are mostly concentrated in 21 Indian provinces. These programmes stated that they admit candidates of but not limited to “graduation in any life sciences”, “3-year bachelor’s degree in any discipline”, “graduation from any Indian universities”, and “graduation in any discipline”. Among the health science disciplines, Modern medicine (*n* = 89; 100%), Occupational therapy (*n* = 57; 64%) is the least eligible. Among the non-health science disciplines, life sciences and behavioural sciences (*n* = 53; 59%) and non-health non-science disciplines, humanities and social sciences (*n* = 62; 72%) are the topmost eligible disciplines for admission in the MPH programmes.

**Conclusion:**

Our review suggests that India’s MlPH programmes are less transdisciplinary. Relatively, non-medical institutions offer admission to various academic disciplines than the medical institutions in their MlPH programmes. India’s Master’s level public health programmes could be more inclusive by opening to graduates from trans-disciplinary backgrounds.

**Supplementary Information:**

The online version contains supplementary material available at 10.1186/s12960-022-00713-4.

## Introduction

Globally, public health education and practice have been transcended and evolved as a transdisciplinary academic discipline [1–7]. The definition of “Transdisciplinarity in Public health” shall be adopted from Stokols et al. “as integrating two or more disciplines, creating fundamentally new conceptual frameworks, perspectives, methods, and synthesizing diverse approaches to address public health problems in real-world settings” [3]. The inter-connections between population health, environment, globalization, climate change and political–demographic–socio-economic–cultural factors demand transdisciplinary research methods and approaches to identify the problems and solutions for the emerging complex global public health problems [1, 8–13]. Globally, public health education and training are rendered as structured, university-affiliated transdisciplinary programmes to systematically nurture the talents and skills of the diversified pool of academic graduates in various countries [14–23]. In the United States of America, United Kingdom, European Union, Australia, New Zealand, Southeast Asia Region, Singapore and China the public health programmes and the institutions were accredited by public health education regulatory bodies with minimum quality standards in terms of inputs, processes, outcomes and performance of the programmes against the evidence-based standards and practice set specific to the nations [24–26].

Historically, public health has evolved as a transdisciplinary profession that exhibits organized collective efforts across multi-sectoral environments traced back to human civilizations’ through the purposeful construction of unique drinking water supplies and sewage drainage systems [27]. In the colonial and pre-independence, diverse geographical regions of the Indian sub-continent, western medicine replaced the indigenous Indian, and Arabic systems of medicine. They influenced the development of health services, medical education mainly to address the prevention and control of epidemics of deadly infectious and endemic tropical diseases. The sole responsibility and focus of the British imperial government were then to alleviate suffering and save people’s lives [28]. Physicians administered the formal public health activities with clinical and public health services. The public health workforce was constituted by personnel from medical (western medicine graduate) and non-medical (allied health sciences graduates and trained in performing specific roles in curative and preventive health services) backgrounds that included auxiliary nurse midwives, nurses, midwives, traditional birth attendants, sanitary inspectors, sanitary assistants, health officers and physicians [27]. Enormous efforts were made to advance the curative and preventive public health services, such as the country’s first-ever Epidemics Prevention Act in 1897, Madras Public Health Act in 1939, hospitals and dispensaries in 1679–1820, medical college hospitals in 1835–1939, Indian Medical Services, vaccination programs, decentralization of health administration with legal provisions to provinces, provincial civic administrative bodies to manage public health, vital statistics and sanitation and establishment of dedicated departments for sanitation and public health [28]. Historically, health was approached predominantly through curative care than the preventive care in the need for immediate relief of ongoing sufferings from diseases. This may have led to the creation of public health cadre fully dominated by physicians trained in western medicine and establishment of medical education, public health courses, such as 3-year Doctor of Medicine (M.D) programmes in Preventive and Social Medicine (PSM), industrial health, safety and hygiene and later on with one or 2-year Diploma in Public Health (DPH) during the post-independence era of Republic of India. Furthermore, these courses were made mandatory for medical doctors to get into public health cadre in the some of the States of Indian Union. In 1995, India’s first Master of Public Health (MPH) programme in the Kottayam University, Kerala and from 1997, the Acheta Menon Centre for Health Science Studies (AMCHSS) of the superspeciality institution at Sree Chitra Tirunal Institute for Medical Sciences & Technology (SCTIMST), Kerala started exclusively to Bachelor of Medicine and Bachelor of Surgery (MBBS) doctors and many institutions followed the same admission criteria across the country.

The necessity of the “Public Health Workforce” and the need for diverse, dedicated, competent public health professionals had never been felt so more critical across the globe than during the ongoing Coronavirus Disease 2019 (COVID-19) crisis [29–33]. The “One Health” concept has become a proof of utility for the transdisciplinary approach to improve surveillance of zoonotic diseases and demonstrated better outcomes through collaborative and transdisciplinary partnerships across multiple sectors of the human–animal–environmental interface [34]. The international community has recognized the need for a transdisciplinary professional workforce in “One health” and included the related concepts in their medical, public health training curriculum and unique MPH degree programmes in One Health [35–38].

According to a review in 2017 by Tiwari et al., public health education is rendered by public and private sectors in medical and non-medical institutions in India, which predominantly includes a 2-year MPH [39]. However, what is not described is the transdisciplinary nature of these programmes based on their admission criteria. In view of the absence of information on trans disciplinarity of India’s Master’s level public health (MlPH) programmes we reviewed, (1) inclusivity aspects by analyzing the programmes’ educational admission criteria, and (2) characterize the programmes by geographical location, type of institutions, course duration, curriculum, pedagogical methods and specializations offered.

## Methods

### Study design

We did a desk review of master’s level public health education programmes in India between December 2020 and August 2021.

### Operational classification

We classified academic educational qualifications into four categories (1) health science, (2) health non-science and, (3) non-health science, (4) non-health non-science disciplines for this study’s scope (Table 1).

**Table 1:** Operational classifications for health science, health non-science and non-health science, non-health non-science academic disciplines

	Science	Non-science
Health	MBBS	
	Bachelor of Dental Surgery (BDS)	
	Bachelor’s degree in AYUSH	
	Physiotherapy and occupational therapy	
	Nursing	
	Pharmaceutical science and studies	
	Animal husbandry and veterinary science	
	Nutrition/dietetics	
	Medical technologies (laboratory, radio-imaging, speech, audiology)	
Non-health	Life sciences	Management studies (public administration, health/hospital administration)
	Agricultural sciences	Planning and development studies (urban, rural)
	Technology/engineering	Law
	Statistics/bio-statistics	Humanities and social studies (anthropology, demography, sociology, social work, population studies, social science)
	Behavioural sciences (psychology)	Journalism, communication
		Economics, commerce

### Developing search strategy

#### Inclusion criteria

We included Master’s level public health programme in the Public health discipline of 2 years ever offered in correspondence/online and on-campus mode during 1995–2021 in India’s public and private sectors.

#### Exclusion criteria

We excluded programmes of less than 24 months of duration, diploma level and 3-year doctorate in medicine (MD) programmes in public health, family medicine, community medicine, public health management community health administration (CHA), preventive and social medicine (PSM), field-epidemiology training programmes (FETP) that does not offer formal degree, certificate courses in public health and allied disciplines, 2-year administration (health and hospital) and management programmes.

#### Search strategy

**Fig. 1:**
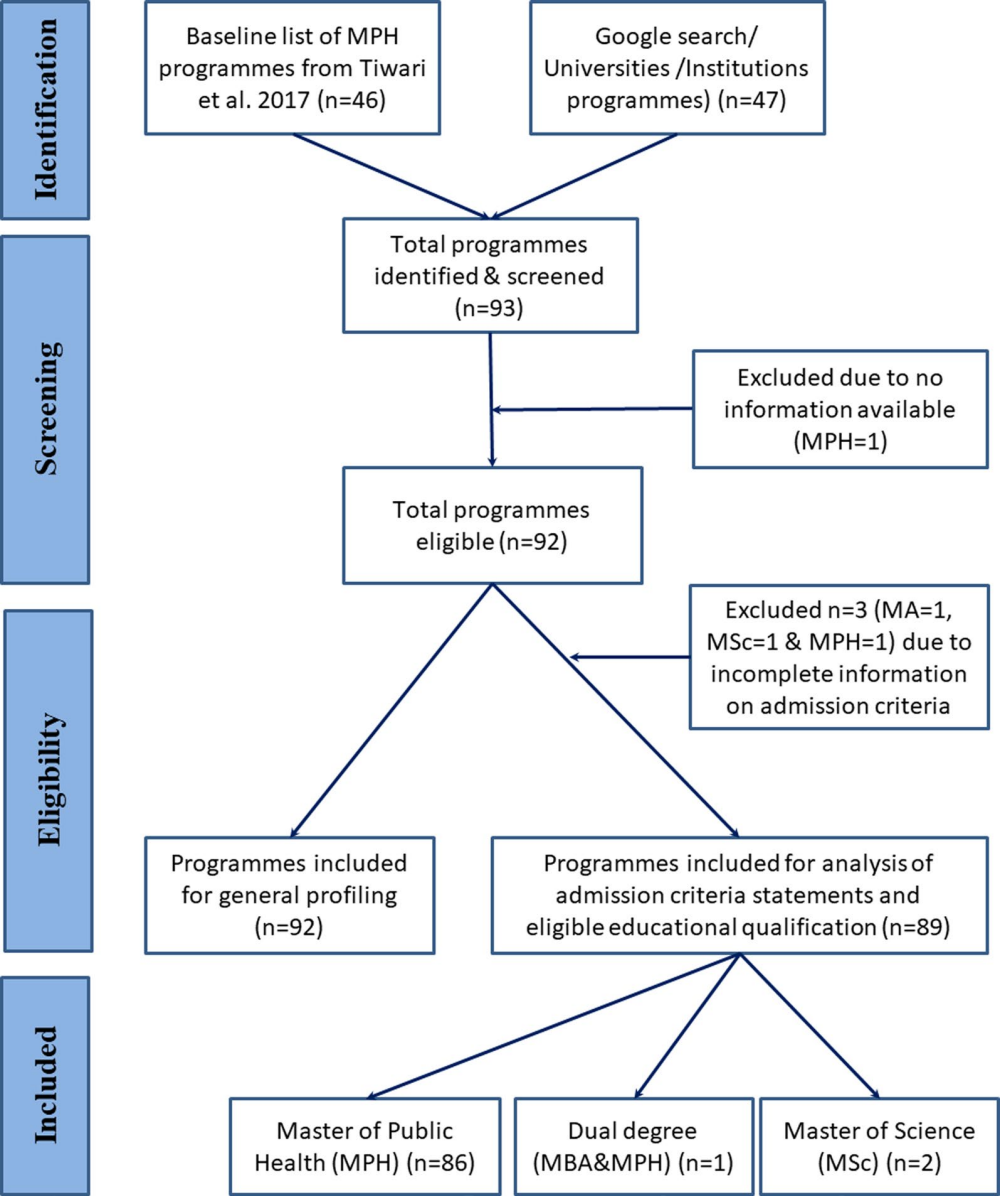
General profile and flow of master’s level public health programmes retrieved, India 1995–2021

Preliminary data regarding the existing MPH programmes were obtained from the review by Tiwari et al. [39] (Fig. 1). To get additional programmes, we used keywords of “master of public health in India”, “public health courses in India”, “public health courses in STATE NAME”, “public health training in India”, “public health education in India”, “MPH”, “schools of public health in India”, “public health colleges in India” and “public health universities in India”. We explored the google search engine through keywords. The preliminary results were used to in-depth searches in the websites of India’s education regulatory bodies, such as the All India Council of Technical Education (AICTE), University Grants Commission (UGC), All India Institute of Medical Sciences (AIIMS), Indian Council of Medical Research (ICMR), professional networking sites (LinkedIn), National Medical Commission (NMC), formerly called ‘Medical Council of India’ and institutions for finding programmes satisfying the inclusion criteria. The list of programmes have been documented, verified through their institutional websites, compared and removed duplicates. Three study investigators were specifically engaged in this activity. We limited our search to public health programmes offered by Indian or any Indo-foreign institutions jointly. We reviewed brochures and prospectus of public health schools and universities in India available on the home pages of the institutions that ever offered master’s degrees in public health discipline. We did not analyze the programmes that do not have adequate information on admission criteria.

### Data abstraction

We abstracted the data in a structured data extraction form. Using our search strategy, we abstracted information regarding each programme on (a) Name of the institution; (b) institution type (public/private and within/outside medical college); (c) affiliated university; (c) geographical location (district and state); (d) course delivery mode [on-campus (full-time/regular) OR correspondence (online/distance) or both]; (e) number of seats; (f) degree specialisation; (g) curriculum and (h) applicants’ educational qualifications eligible for admission into the course.

### Analysis plan

We calculated descriptive statistics for the admissible educational qualifications for the programmes as per our operational classifications. Furthermore, we profiled the programmes in terms of geographical location, type of institutions, course duration, curriculum, pedagogical methods and specializations offered. We used an open-source geographic information system QGIS version 3.0.3 software and two geography data layers (ne_50m_admin_0_countries, ne_50m_admin_0_disputed_areas) from Natural Earth open source website and generated a spot map to present the distribution of the programmes [40]. We counted the number of programmes and treated each specialization degree as a unique programme irrespective of the same institution offering in the same or multiple geographies.

## Results

### Public health programmes identified and included

We could identify 92 programmes (Fig. 1). There were 87 MPH, three Master of Science (M.Sc.), one dual-degree MPH with Master of Business Administration (MPH/MBA) and one Master of Arts (MA) degree programme by 76 institutions (Medical schools—21 and Non-medical schools—55) or universities. Additional file 1: Table S1 shows this in more detail.

### Descriptive profile of the public health programmes

#### Geographical distribution

**Fig. 2:**
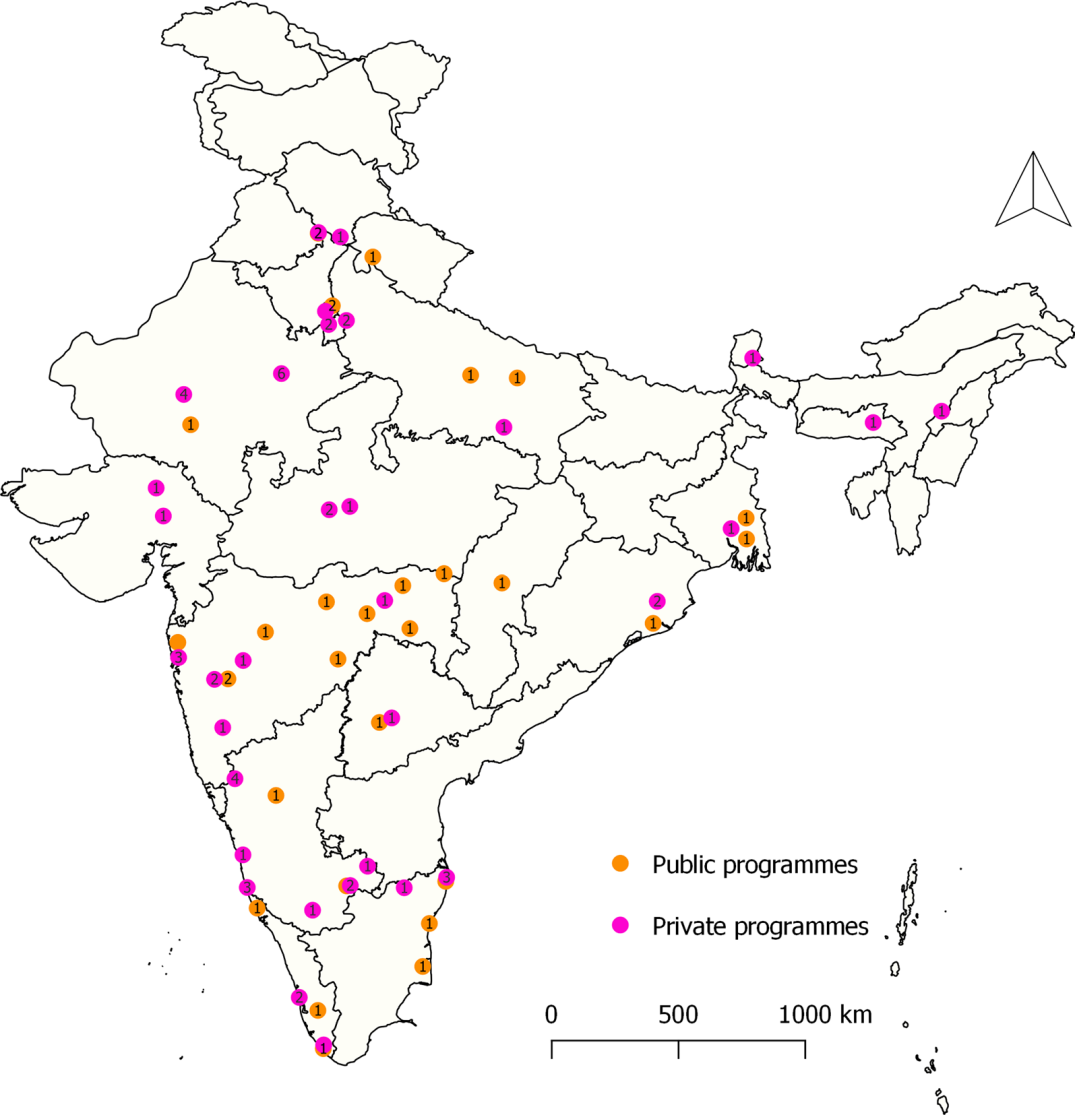
Distribution of public and private master’s level public health programmes, India 1995–2021 (*n* = 92)

Of the 92 programmes, 58 (63%) are offered in private institutions. Additional file 1: Table S2 shows this in more detail. All these programmes are concentrated in 21 States and Union territories of India. The majority of the programmes, 32 (35%), are offered in the western region, followed by 31 (34%) programmes in India’s southern region (Additional file 1: Table S2). Of 34 programmes provided by public institutions, 23 (68%) are located in southern and western regions. No government public health programmes are offered in the Northeast states (Fig. 2). Of 58 private programmes, 12 (21%) are in Karnataka, followed by 10 (17%) in Rajasthan (Fig. 2).

#### Duration of the programme

Of the 92 programmes offered, 90 are of 2-year duration and two 3-year programme at the Rajiv Gandhi University of Health Sciences (RGUHS), Karnataka and All India Institute of Hygiene and Public Health (AIIH&PH), West Bengal, one of the oldest institution offering public health training programmes.

#### Specializations offered

Of 76 institutions, 20 (26%) institutions (Public = 13 and Private = 7) offered 29 specialization degree programmes in public health domains, such as Epidemiology, Social Epidemiology, Field Epidemiology, Global Health, Maternal and Child Health, Surveillance and Epidemics, Nutrition, Health Economics, Policy and Financing, Environmental and Occupational Health, Health Systems Management and Administration, Quality and Safety, Digital Health, Public Health Informatics. One institution offers a dual degree in Public health combined with an MBA.

#### Pedagogical methods

Of 92 programmes, two were distance learning offered by two institutions (Global Open University, Nagaland; Datta Meghe Institute of Medical Sciences, Maharashtra). Two institutions [Chitkara University and the Asian Institute of Public Health University (AIPHU)] offered two tracks of MPH course in collaboration with a foreign University [University of Nebraska Medical Center (UNMC), USA]. This programme has the option to obtain the degree either in India or abroad. The remaining 90 master’s programmes in public health are taught on-campus. The course work covers standard public health fields, including epidemiology, biostatistics, environmental health, health policy and finance. Most on-campus programmes include one and half years of classroom learning and 3–6 months of practical/field experience, capstone dissertation projects.

### Admission criteria of the public health programmes

#### Nature of statement of admissibility criteria

The eligibility criteria for master’s public health programmes are variable and vague; for example, some institutions enrol graduates with a 3-year degree, while some enrol 4-year degrees; in contrast to some institutions admitting undergraduate (UG) health science graduates and postgraduate (PG) degrees for non-health science graduates. Some institutions accept graduates of any science stream or non-science stream or any discipline graduated from UGC recognized universities. Eligibility criteria do not differ for the MlPH specialization degrees programmes offered by the same institution.

Some institutions prefer candidates with prior experience in the health services and public health field. Twenty institutions (Public—17 and Private—3) offer programmes for sponsored candidates from the respective state governments and non-governmental organizations. Three public institutions out of 34 exclusively enrol in-service MBBS graduates funded by the various sub-national health departments. Other 19 institutions have 5–60% reservation in their MlPH programmes for candidates from the state government, bi-lateral organizations with sponsorships and their employees/minority quota. One private institution offers an executive MPH for in-service medical and dental graduates professionals placed in public and private sectors.

#### Distribution of the admission eligibility by academic disciplines

Among the health science disciplines, all the programmes offered for the Medicine graduates (*n* = 89; 100%) (Table 2). Occupational therapy (*n* = 57; 64%) is the least eligible. Among the non-health science disciplines, life sciences and behavioural sciences (*n* = 53; 59%) and non-health non-science disciplines, humanities and social sciences (*n* = 62; 72%) are the topmost eligible disciplines for admission in the MlPH programmes.

**Table 2:** Number and proportion of various academic disciplines eligible for admission in master’s level public health programmes, India 1995–2021

Academic disciplines	Programme type				
	Private (*n* = 55)		Public (*n* = 34)		Total
	Medical college	Non-medical college	Medical college	Non-medical college	
	*n* = 7	*n* = 48	*n* = 15	*n* = 19	*n* (%)
Health science					
Medicine	7	48	15	19	89 (100)
Dentistry	7	48	14	17	86 (97)
Nursing	6	47	14	17	84 (94)
AYUSH	6	47	14	15	82 (92)
Physical therapy	5	45	14	15	79 (89)
Medical technologies	4	41	12	14	71 (80)
Pharmaceutical science and studies	6	44	5	15	70 (79)
Nutrition/dietetics	5	41	5	12	63 (71)
Animal husbandry and veterinary science	4	38	5	13	60 (67)
Occupational therapy	4	35	5	13	57 (64)
Non-health science					
Life sciences	3	35	2	13	53 (59)
Behavioural sciences	3	35	2	13	53 (59)
Technology and engineering	3	33	5	7	48 (54)
Statistics and bio-statistics	2	21	3	9	35 (39)
Agricultural sciences	0	23	2	6	31 (35)
Non-health and non-science					
Humanities and social sciences	5	37	5	15	62 (70)
Economics and commerce	2	28	3	7	40 (45)
Management studies	2	25	3	6	36 (40)
Law	1	27	3	4	35 (39)
Journalism and communications	0	2	0	1	3

## Discussion

We reviewed the admission criteria of India’s Master’s level public health programmes and identified that they are less transdisciplinary. Despite a doubling of these programmes in the recent 5 years, we determined that they mostly catered to the medical and allied health disciplines and offered limited scope for non-health, academic disciplinary graduates.

### Evolution of MPH programmes and public health schools in India

Our findings are the results of highly medicalized public health programmes which has its origins in the historical context of the India’s pre-independence era [41]. To impart public health education in India, the School of Tropical Medicine in 1912 and the AIIH&PH in 1932, was created to teach public health out of medical colleges. This was a conscious shift made to avoid conflicts and tensions between public health academicians and clinicians; apprehension of the American experience through Welch-Rose Report 1915 resulted in drifting apart of Public health and Medicine disciplines into two separate institutions [42]. The Mudaliar committee in 1959 recommended for establishment of Schools of Public Health in every sub-national region and rendered public health education to both MBBS and non-MBBS personals to create multi-disciplinary public health professionals [43]. However, ironically, post-independence in the Union of India, Public Health education was continued to be offered only in the medical colleges. The teaching was one of the curricula in the undergraduate program and 2-year diploma, 3-year postgraduate speciality degrees, considering that public health and medicine have been mutually interdependent. The decision-makers presumed that medicine graduates shall only address public health. The majority of the State machinery was depended on the training of in-service medical officers and continued fostering their fraternities in national, sub-national public health departments for administrative positions [42].

The expert committee on Public Health Systems during 1995–97 created the impetus for establishing the first two MPH programmes in the state of Kerala [43]. From 1995 to 2016, 44 institutions commenced MlPH programmes predominantly in private sectors, understanding and accepting this field’s transdisciplinary nature may have led to the doubling of Public health institutions and programmes between 2016 and 2021. From 2005 to 2006 onwards, many private institutions started offering the MlPH courses to both health and allied health science graduates and slowly expanded to include non-health science, technical and arts disciplines. From 2013 onwards, many private and state institutions also started offering 3-year bachelor degree programmes in public health (BPH); exceptionally, RGUHS in the State of Karnataka offers a 4-year bachelor programme in Public health from 2019 to 2020. Till 2019, eight institutions across India offered BPH programmes [44]. As these institutions are mostly outside the medical schools, they encourage non-health and social sciences and arts academic graduates to enrol in their master’s level public health programmes.

The degree of inclusivity involving non-health, allied health sciences and non-science academic graduates varies between public and private institutions. We find that private institutions offer a broader scope for graduates of trans-disciplinary educational backgrounds to get trained in the public health discipline than public institutions. Undeniably, scenarios are encouraging in public institutions the second MPH programme established in SCTIMST, Kerala moved away from MBBS doctor centric admission from 2011 onwards and included graduates from health, allied health sciences, social sciences and nutrition graduates in their admission, from 2015 onwards opened its door to wide-array of academic disciplines mentioned in this study [45, 46].

Unlike in other countries, in the Indian subcontinent, establishment processes of public health schools and programmes are less complicated and well-supported with financial, technical resources, political, and institutional support, as proved through a surge of programmes in the private sector, including undergraduate public health degree programmes [26, 44, 47, 48]. Simultaneously, these private institutions should maintain the scholars’ adequate ‘standard’ and quality and provide them with the knowledge, skills, and abilities necessary to become competent professionals to meet the needs of contemporary public health challenges and issues facing India and globally [49–51].

### Indian MlPH programmes in the global context

The teaching hours ranging from 50 to 120 credit hours, curriculum, pedagogical methods, admission processes of the Indian MlPH programmes are not uniform and highly variable across the institutions. There is currently no formal body for accrediting and standardization of public health programmes and institutions and maintaining uniform quality education standards, a scenario similar to the state of MPH programmes in middle-eastern countries [23]. There are no knowledge-sharing forums, platforms, or networks between the various schools of public health and programmes. The NMC recognized some of the programmes offered in public institutions, and many programmes are not. Due to the limited operability functions of the NMC website, we couldn’t retrieve this information.

Some of the pioneer schools of public health in the Indian subcontinent have much greater standardization of curriculum specific to their operations and expertise in the epidemiological and implementation research, programme implementation and advocacy, health system strengthening, socio-economic and political determinants of health, caste, gender and indigenous population, health technologies and health systems resources. We couldn’t do an in-depth analysis of the curriculum, admission processes, university affiliation and comparison of the institutes due to the lack of information on these aspects in public domains for most of the programmes and institutions.

Most of the institutions offering MlPH courses have adopted in total or parts of the comprehensive “model course curriculum for MPH” guidelines released in 2018 by the Ministry of Health and Family Welfare, the Union Government of India [52]. While the curriculum acknowledges the trans-disciplinary nature of public health and the need to produce competent transdisciplinary public health professionals comprising science and Non-science graduates, it fails to delineate the definition of ‘science’ and ‘non-science’ academic disciplines [52]. The Skills and Values mentioned therein does not necessarily indicate the need for any particular educational qualification in line with the course eligibility criteria; however, the four broad competencies and the description of the “importance of professionalism, values and communication”, is excessively “medical” centric and targeted towards bio-medicine qualified professionals.

### Professional identity crisis

There was limited information available in the public domains on the number of admission capacities, the number of candidates admitted, categories of academic graduates admitted in the programmes and the number of candidates successfully passed out each year from these 92 institutions. Being rendered mainly as a postgraduate course, bachelor and master degree holders from different educational qualifications get enrolled and exit every year. Due to lack of national policy for public health professional registration, practice and limited scope for the enrollment in professional bodies, such as the Indian Association of Preventive and Social Medicine (IAPSM), and Indian Public Health Association (IPHA), the trans-disciplinary public health professionals lack an innate identity for themselves in India [24, 39, 53].

### Employment opportunities

India’s National Health Policy 2017 paved the road-map for establishing public health management cadres and appropriate career structure for the multi-disciplinary public health professionals in the national and sub-national health departments [54]. This vision was not materialized at the federal and provincial levels [55–58]. Parallelly, non-MBBS public health professionals were engaged as contractual staff in various national health programmes, national and sub-national health systems resource centres, federal and provincial health ministries and departments, bilateral organizations, private sectors, non-governmental and non-profit organizations and health research organizations in various capacities [59, 60]. Nevertheless, in the last two decades until 2019, non-MBBS public health graduates have also been considered for regular positions in health research organizations of national importance.

Public health education practices in the Indian subcontinent require reform to shape the public health profession [49, 50, 61–64]. India’s model course curriculum for MPH programmes also stressed the need for transdisciplinary public health professionals who have a basic understanding of the various determinants of health. Currently, public health programmes are skewed towards India’s two regions, barring backward states with a huge burden of poverty and diseases of public health importance despite the need for a transdisciplinary public health workforce [65, 66]. Furthermore, the production of transdisciplinary public health professionals also demands national and provincial policies to ensure the relevance of these professionals, defined career pathway and equal employment opportunities in health and non-health sectors in the Indian subcontinent. India’s heterogeneous health care system, underdeveloped public health governance, vertical disease control and health service delivery programmes necessitate the emergence of a cadre of competent professionals specially trained in public health [61, 67]. Nevertheless, implementing a comprehensive One Health concept for addressing contemporary global health issues necessitates collaborative, coordinated, concerted actions across sectors and various disciplines, which can be achieved by having a transdisciplinary public health workforce [68, 69]. Thus, public health training in low and middle-income countries (LMICs) should be inclusive and foster the transdisciplinary public health workforce and their competencies across the health and non-health sector to help achieve the nation’s health goals.

## Limitations

Our study included only the programmes for which information was available in the public domain, such as prospectus, brochures and websites. We could not capture the changes in admission criteria for MlPH programmes except for SCTIMST, Kerala. We captured a maximum number of programmes of pre-eminent public and private institutions that have ever offered MlPH in India through our search strategy. We believe that we would not have missed any programme in our search strategy. Second, we could not directly verify the information with the respective institutions to confirm whether programme admission criteria are operational and remain unchanged for the current academic year. However, most of the programmes had their updated homepage at the time of review and hence, we think any misclassification is unlikely.

## Conclusions and recommendations

On the basis of our findings, we conclude that India’s master’s level public health programmes are less transdisciplinary in nature. We recommend that they become more inclusive and are offered to students from diverse academic backgrounds to produce a competent transdisciplinary public health workforce to meet the needs of India’s human resources for health. Such graduates can potentially contribute to a resilient health care system that can meet future health challenges, including pandemics. This needs to be supported by policy level endorsement at various levels for considering such graduates for positions at various levels of the public or private systems.

## Supplementary Information

**Additional file 1: Table S1.** List of universities/institutions that offer master’s level public health programmes, India 1995–2021 (in alphabetical order *n* = 76 institutions and 92 programmes). **Table S2.** Geographical distribution of public health master’s programme by type of institutions, India 1995–2021.

## Data Availability

All data generated or analyzed during this study are included in this published article and its Additional files.

## Abbreviations

MlPH: Master’s-level Public Health
MBBS: Bachelor of Medicine and Bachelor of Surgery
BDS: Bachelor of Dental Surgery
AYUSH: Ayurveda, Yoga and Naturopathy, Unani, Siddha/Sowa-Rigpa and Homeopathy
CHA: Community Health Administration
PSM: Preventive and Social Medicine
FETP: Field-epidemiology Training Programmes
AICTE: All India Council of Technical Education
UGC: University Grants Commission
GIS: Geographic Information System
MPH: Master of Public Health
MSc: Master of Science
MA: Master of Arts
MBA: Master of Business Administration
UNMC: University of Nebraska Medical Centre
UG: Undergraduate
PG: Postgraduate
IAPSM: Indian Association of Preventive and Social Medicine
IPHA: Indian Public Health Association
LMIC: Low and Middle Income Country
