# Supplementary material for: Transdisciplinarity of India’s master’s level public health programmes: evidence from admission criteria of the programmes offered since 1995
Source: Hum Resour Health. 2022 Feb 2;20:14. doi: 10.1186/s12960-022-00713-4 (PMC8809628; doi:10.1186/s12960-022-00713-4)
Supplement: Supplementary file 1 — Additional file 1: Table S1. List of universities/institutions that offer master’s level public health programmes, India 1995–2021 (in alphabetical order n = 76 institutions and 92 programmes). Table S2. Geographical distribution of public health master’s programme by type of institutions, India 1995–2021. [file 12960_2022_713_MOESM1_ESM.docx]

**Table S1**: List of universities/institutions that offer master's level public health programmes, India 1995 – 2021 (in alphabetical order n=76 institutions and 92 programmes)

| **S.no** | **Name of the Institutions/Universities and their location** | **Number of Master's Public Health Programmes** | | |
| --- | --- | --- | --- | --- |
|  |  | **Private** | **Public** | **Total** |
| 1 | Achutha Menon Centre for Health Science Studies SCTIMST, Thiruvananthapuram, Kerala |  | 1 | 1 |
| 2 | All India Institute of Hygiene and Public Health, Kolkatta, West Bengal |  | 1 | 1 |
| 3 | All India Institute of Medical Sciences, Jodhpur, Rajasthan |  | 1 | 1 |
| 4 | All India Institute of Medical Sciences, Rishikesh, Uttarakhand |  | 1 | 1 |
| 5 | All India Institute of Medical Sciences, Raipur, Chhattisgarh |  | 1 | 1 |
| 6 | Amity University, Noida, Uttar Pradesh | 1 |  | 1 |
| 7 | Amrita Institute of Medical Sciences and Research Centre, Kochi, Kerala | 2 |  | 2 |
| 8 | Asian Institite of Public Health University, Bhubaneswar, Odisha | 1 |  | 1 |
| 9 | B J Government Medical College, Pune, Maharashtra |  | 1 | 1 |
| 10 | Central University of Kerala, Kasargode |  | 1 | 1 |
| 11 | Central University of Tamil Nadu, Thiruvarur |  | 1 | 1 |
| 12 | Chitkara University, Chandigarh | 1 |  | 1 |
| 13 | Christian Medical College, Vellore, Tamil Nadu | 1 |  | 1 |
| 14 | Datta Meghe Institute of Medical Sciences, Wardha, Maharashtra | 1 |  | 1 |
| 15 | Delhi Pharmaceutical Sciences and Research University, Pushp Vihar, New Delhi | 1 |  | 1 |
| 16 | Dr Shankarrao Chavan Government Medical College, Nanded, Maharashtra |  | 1 | 1 |
| 17 | Dr. Rammanohar Lohia Avadh University, Ayodhya, Uttar Pradesh |  | 1 | 1 |
| 18 | Edward & Cynthia Institute of Public Health, Mangalauru, Karnataka | 1 |  | 1 |
| 19 | Eternal University, Sirmour, Himachal Pradesh | 1 |  | 1 |
| 20 | Global Institute of Healthcare Management, Najafgarh, Delhi-National Capital Region | 1 |  | 1 |
| 21 | Global Institute of Public Health, Thiruvananthapuram, Kerala | 1 |  | 1 |
| 22 | Government Medical College, Akola, Maharashtra |  | 1 | 1 |
| 23 | Government Medical College, Aurangabad, Maharashtra |  | 1 | 1 |
| 24 | Government Medical College, Chandrapur, Maharashtra |  | 1 | 1 |
| 25 | Government Medical College, Gondia, Maharashtra |  | 1 | 1 |
| 26 | Government Medical College, Nagpur, Maharashtra |  | 1 | 1 |
| 27 | Grant Government Medical College, Mumbai, Maharashtra |  | 1 | 1 |
| 28 | ICMR - National Institute of Epidemiology, Chennai, Tamil Nadu |  | 1 | 1 |
| 29 | ICRI - Sam Global University, Bhopal, Madhya Pradesh | 1 |  | 1 |
| 30 | Indian Institute of Health Management Research, Jaipur, Rajasthan | 1 |  | 1 |
| 31 | Indian Institute of Public Health - Delhi-National Capital Region | 1 |  | 1 |
| 32 | Indian Institute of Public Health – Gandhinagar, Gujarat | 1 |  | 1 |
| 33 | Indian Institute of Public Health – Hyderabad, Telangana | 1 |  | 1 |
| 34 | Indian Institute of Public Health – Shillong, Meghalaya | 1 |  | 1 |
| 35 | Institute of Public Health, Kalyani, West Bengal |  | 1 | 1 |
| 36 | Interdisciplinary School of Health Sciences, Savitribai Phule Pune University, Pune, Maharshtra |  | 1 | 1 |
| 37 | Jagadguru Sri Shivarathreeswara University, Mysuru, Karnataka | 1 |  | 1 |
| 38 | Jawaharlal Institute of Postgraduate Medical Education and Research, Puducherry |  | 1 | 1 |
| 39 | Jawaharlal Nehru University, Munirka, New Delhi |  | 1 | 1 |
| 40 | Jodhpur School of Public Health, Jodhpur, Jaipur Rajasthan | 8 |  | 8 |
| 41 | Kalinga Institute of Industrial Technology University, Bhubaneswar, Odisha | 1 |  | 1 |
| 42 | Karnatak Lingayat Education Academy of Higher Education and Research, Belgaum, Karnataka | 4 |  | 4 |
| 43 | Karnataka State Rural Development & Panchayat Raj University, Gadag, Karnataka |  | 1 | 1 |
| 44 | KPC Medical College, Kolkatta, West Bengal | 1 |  | 1 |
| 45 | Krishna Institute of Medical Sciences, Karad, Maharashtra | 1 |  | 1 |
| 46 | Mahatma Gandhi University, Kottayam, Kerala |  | 1 | 1 |
| 47 | Mahatma Jyoti Rao Phoole University, Jaipur, Rajasthan | 1 |  | 1 |
| 48 | Mansarovar Global University, Sehore, Madhya Pradesh | 2 |  | 2 |
| 49 | MD Goenka University, Sohna, Haryana | 1 |  | 1 |
| 50 | MIT World Peace University, Pune, Maharashtra | 1 |  | 1 |
| 51 | MS Ramaiah University of Applied Sciences, Bengaluru, Karnataka | 1 |  | 1 |
| 52 | National Centre for Disease Control, Shamnath Marg, New Delhi |  | 1 | 1 |
| 53 | National Institute of Mental Health and Neuro Sciences, Bengaluru, Karanataka |  | 1 | 1 |
| 54 | National Institute of Public Health Training & Reearch |  | 1 | 1 |
| 55 | Nitte University - KS Hegde Medical Academy, Mangaluru, Karnataka | 1 |  | 1 |
| 56 | Noida International University, Gautham Budh Nagar, Uttar Pradesh | 1 |  | 1 |
| 57 | Padmashree School of Public Health, Bengaluru, Karnataka | 1 |  | 1 |
| 58 | Panjab University, Chandigarh |  | 1 | 1 |
| 59 | Parul University, Ahmedabad, Gujarat | 1 |  | 1 |
| 60 | Post Graduate Institute of Medical Education and Research, Chandigarh |  | 1 | 1 |
| 61 | Prasanna School of Public Health Manipal University, Manipal, Karnataka | 1 |  | 1 |
| 62 | Pravara Institute of Medical Sciences, Ahmed Nagar, Maharashtra | 1 |  | 1 |
| 63 | Rajiv Gandhi University of Health Sciences, Bengaluru, Karnataka |  | 1 | 1 |
| 64 | Shalom Institute of Health & Allied Sciences SHUATS, Allahabad, Uttar Pradesh | 1 |  | 1 |
| 65 | Sri Devaraj Urs Academy of Higher Education and Research, Kolar, Karnataka | 1 |  | 1 |
| 66 | Sri Ramachandra Medical College and Research Institute, Chennai, Tamil Nadu | 1 |  | 1 |
| 67 | Sri Ramaswamy Memorial Institute of Science and Technology, Chennai, Tamil Nadu and Gangtok, Sikkim | 3 |  | 3 |
| 68 | Symbiosis Institute of Health Sciences, Pune, Maharashtra | 1 |  | 1 |
| 69 | Tata Institute of Social Sciences, Mumbai, Maharashtra | 3 |  | 3 |
| 70 | The Global Open University, Dimapur, Nagaland | 1 |  | 1 |
| 71 | The Tamil Nadu Dr. M.G.R. Medical Univeristy, Chennai, Tamil Nadu |  | 1 | 1 |
| 72 | University of Hyderabad, Telangana |  | 1 | 1 |
| 73 | University of Lucknow, Uttar Pradesh |  | 1 | 1 |
| 74 | Utkal University, Bhubaneswar, Odisha |  | 1 | 1 |
| 75 | Vasantrao Naik Government Medical College, Yavatmal, Maharashtra |  | 1 | 1 |
| 76 | Yenepoya Medical College, Mangaluru, Karnataka | 1 |  | 1 |
|  | Total | 58 | 34 | 92 |

**Table S2**: **Geographical distribution of public health master's programmes by type of institutions, India 1995-2021**

| **Distribution of Public health master's programme by type of institutions** | | | |
| --- | --- | --- | --- |
| **Regions (States)** | **Private n (%)** | **Public n (%)** | **Total n (%)** |
| North (Chandigarh, Delhi, Haryana, Himachal Pradesh) | 6 (10) | 5 (15) | 11 (12) |
| South (Karnataka, Kerala, Puducherry, Tamil Nadu, Telangana) | 20 (34) | 11 (32) | 31 (34) |
| Central (Chhattisgarh, Madhya Pradesh, Uttar Pradesh) | 6 (10) | 3 (9) | 9 (10) |
| Northeast (Meghalaya, Nagaland, Sikkim) | 3 (5) | 0 | 3 (3) |
| East (Odisha, West Bengal) | 3 (5) | 3 (9) | 6 (6) |
| West (Gujarat, Maharashtra, Rajasthan) | 20 (34) | 12 (35) | 32 (35) |
| Total n (%) | 58 (63) | 34 (37) | 92 (100) |
